# What is the effect of a combined physical activity and fall prevention intervention enhanced with health coaching and pedometers on older adults’ physical activity levels and mobility-related goals?: Study protocol for a randomised controlled trial

**DOI:** 10.1186/s12889-015-1380-7

**Published:** 2015-05-09

**Authors:** Anne Tiedemann, Serene Paul, Elisabeth Ramsay, Sandra D O’Rourke, Kathryn Chamberlain, Catherine Kirkham, Dafna Merom, Nicola Fairhall, Juliana S Oliveira, Leanne Hassett, Catherine Sherrington

**Affiliations:** The George Institute for Global Health, Sydney, Australia; The University of Sydney, Camperdown, Australia; Neuroscience Research Australia, Randwick, Australia; University of Western Sydney, Penrith, Australia

**Keywords:** Physical activity, Exercise, Prevention, Aged, Health coaching, Intervention studies, Accidental falls, Clinical trial, Pedometer

## Abstract

**Background:**

Physical inactivity and falls in older people are important public health problems. Health conditions that could be ameliorated with physical activity are particularly common in older people. One in three people aged 65 years and over fall at least once annually, often resulting in significant injuries and ongoing disability. These problems need to be urgently addressed as the population proportion of older people is rapidly rising. This trial aims to establish the impact of a combined physical activity and fall prevention intervention compared to an advice brochure on objectively measured physical activity participation and mobility-related goal attainment among people aged 60 +.

**Methods/design:**

A randomised controlled trial involving 130 consenting community-dwelling older people will be conducted. Participants will be individually randomised to a control group (n = 65) and receive a fall prevention brochure, or to an intervention group (n = 65) and receive the brochure plus physical activity promotion and fall prevention intervention enhanced with health coaching and a pedometer.

Primary outcomes will be objectively measured physical activity and mobility-related goal attainment, measured at both six and 12 months post randomisation. Secondary outcomes will include: falls, the proportion of people meeting the physical activity guidelines, quality of life, fear of falling, mood, and mobility limitation. Barriers and enablers to physical activity participation will be measured 6 months after randomisation.

General linear models will be used to assess the effect of group allocation on the continuously-scored primary and secondary outcome measures, after adjusting for baseline scores. Between-group differences in goal attainment (primary outcome) will be analysed with ordinal regression. The number of falls per person-year will be analysed using negative binomial regression models to estimate the between-group difference in fall rates after one year (secondary outcome). Modified Poisson regression models will compare groups on dichotomous outcome measures. Analyses will be pre-planned, conducted while masked to group allocation and will use an intention-to-treat approach.

**Discussion:**

This trial will address a key gap in evidence regarding physical activity and fall prevention for older people and will evaluate a program that could be directly implemented within Australian health services.

**Trial registration:**

ACTRN12614000016639, 7/01/2014.

## Background

Physical inactivity is estimated to cause more than five million deaths worldwide each year as reported in the *Lancet* special issue on physical activity [1]. Physical inactivity is particularly common in older age [2]. Experiencing a fall in older age is also a significant and common public health issue that can result in substantial injury and ongoing disability [3]. At least one third of people aged 65 years and over fall once or more annually [4].

Systematic reviews show that well-designed, structured exercise programs are effective in preventing falls in community-dwelling older people [5,6]. Exercise is most effective if it challenges balance [6]. However, exercise interventions found to be effective for falls prevention [5,6] have not generally been of a high enough dose to ensure participants also meet physical activity recommendations and obtain broader health benefits. Furthermore, several intervention programs aimed at increasing physical activity levels among older people have actually increased falls. A trial by Ebrahim et al. [7] involving 165 women with a history of recent upper limb fracture found that a brisk outdoor walking program significantly increased fall rates. Similarly in a large-scale trial (n = 1089) conducted by Lawton et al. [8] among women aged 40–75 years, physical activity prescription with telephone support successfully increased physical activity levels but also increased falls (p < 0.001) and injuries (p = 0.03). These results suggest that physical activity programs for older adults should include fall prevention components.

This trial aims to establish the impact of a physical activity and fall prevention intervention compared to an advice brochure on older people’s physical activity levels, mobility-related goal attainment, fall rates, quality of life, fear of falling, mood and mobility limitation.

## Methods

### Trial design

**Figure 1:**
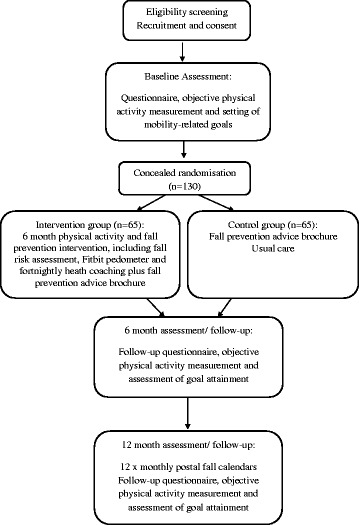
Trial design.

We will conduct a randomised controlled trial. The design of the trial is illustrated in Figure 1. This trial has been designed according to the CONsolidated Standards Of Reporting Trials (CONSORT) statement [9] and is reported according to the Standard Protocol Items: Recommendations for Interventional Trials (SPIRIT) statement [10] and with reference to the Template for Intervention Description and Replication (TIDieR) checklist [11].

### Participants

130 consenting community-dwelling people aged 60 years and over will be randomised to either the intervention group (fall prevention advice brochure plus physical activity promotion and fall prevention intervention) or a control group (fall prevention advice brochure only). People will be eligible for inclusion in the trial if they are aged 60+ years; living at home; regular (weekly) users of the internet via a computer or tablet device; leave the house regularly (at least once per week) without physical assistance from another person.

Potential participants will be excluded from participation if they: are “house-bound” (not having gone outside without physical assistance from another person in the past month); have a cognitive impairment (a diagnosis of dementia or a Memory Impairment Screen [12] score of less than 5); have insufficient English language skills to fully participate in the program; have a progressive neurological disease (e.g., Parkinson’s disease); have a medical condition precluding exercise (e.g., unstable cardiac disease); already meet the Australian Physical Activity Guidelines for older adults [13] (operationalised as 150 minutes of moderate intensity physical activity per week, assessed using the Incidental and Planned Exercise Questionnaire (IPEQ [14])) and have had a falls risk assessment in the past year, since they may already be receiving the fall prevention intervention. If it is unclear whether a potential participant meets the eligibility criteria his/her permission will be sought to discuss this with a family member or health care professional (e.g. General Practitioner).

### Recruitment and randomisation

Participants will be recruited in metropolitan Sydney and surrounds, in Australia via community-based newspaper advertisements, local council websites, and newsletters and mailing lists of established organisations for older people, commencing in January 2014. Allocation to study groups will take place after completion of baseline questionnaires, the assessment of current physical activity participation and the setting of mobility-related goals. To ensure allocation concealment, randomisation to groups will be undertaken by an investigator not involved in recruitment using a computer generated random number schedule with randomly permuted block sizes of between 2 and 6.

### Intervention group

Participants allocated to the intervention group (n = 65) will receive the “Staying Active and On Your Feet” booklet developed by the NSW Ministry of Health and will receive an intervention aimed at increasing physical activity participation and reducing risk of falling. It involves administration of the *QuickScreen* fall risk assessment [15], implementation of tailored fall prevention strategies and a physical activity plan. Health coaching will be used to identify barriers and facilitators to physical activity participation, and to provide education and support to assist participants to achieve their physical activity goals.

A pedometer enhanced with a web-interface (“fitbit”, www.fitbit.com/au) will be provided to all intervention group participants to give feedback on the amount of daily physical activity achieved. The *fitbits* will also be provided as a motivational tool to encourage ongoing physical activity participation. The intervention will be delivered during one home visit, lasting approximately two hours, by a health coach with a professional background in physiotherapy. The health coach will also be in contact with intervention participants via telephone or email every two weeks, for a total period of 6 months, to monitor and facilitate progress towards physical activity goals and to assist participants to overcome any participation barriers that arise. During the bi-weekly telephone contact, health coaches will also enquire about the circumstances of any falls that participants may have experienced and they will discuss strategies for reducing the risk of future falls. Intervention participants will also be assisted to find suitable local exercise opportunities (e.g. Tai Chi, balance and strength training) that will be identified using the NSW Ministry of Health’s *Active and Healthy* online database (http://www.activeandhealthy.nsw.gov.au/).

Participants will be encouraged to wear the pedometer during waking hours on a daily basis for the whole 6 month intervention period to record their daily steps and provide feedback and motivation to increase their physical activity participation. The *fitbit* enhanced pedometer is designed to wirelessly synchronise with computer software to download stored physical activity information. Participants will be encouraged to synchronise and download their data on a weekly basis or more often if desired. During the home visit to implement the intervention, participants will be taught how to use the *fitbit* device and the associated internet based feedback and monitoring technology. The research team will have access to all intervention participants’ *fitbit* data and will monitor individual adherence with the intervention. If participants have not uploaded their *fitbit* data to their computer or internet-connected tablet device in the past week, during the fortnightly contact their health coach will enquire about any problems encountered with the pedometer and they will encourage participant compliance with the intervention protocol. Table 1 summarises the intervention content.

**Table 1:** **Intervention description using the Template for Intervention Description and Replication (TIDieR) checklist**

1. Brief name	Combined physical activity promotion and fall prevention intervention enhanced with health coaching and pedometers to increase older adults’ physical activity levels and mobility-related goals.
2. Why	Physical inactivity and falls in older people are important public health problems. Health conditions that could be ameliorated with physical activity are particularly common in older people. One in three people aged 65 years and over fall at least once annually, often resulting in significant injuries and ongoing disability. These problems need to be urgently addressed as the population proportion of older people is rapidly rising.
3. What- materials	Participants will receive:
	•The “Staying Active and On Your Feet” fall prevention booklet developed by the NSW Ministry of Health
	•An assessment of their fall risk factors using the *QuickScreen* fall risk assessment [15]
	•A pedometer enhanced with a web-interface ("fitbit", www.fitbit.com/au) to give feedback on the amount of daily physical activity achieved.
4. What- procedures	Telephone or email-based health coaching will be used to identify barriers and facilitators to physical activity participation, and to provide education and support to assist participants to reduce their risk of falling and to achieve their physical activity goals.
5. Who provided	Three health coaches with professional backgrounds in physiotherapy will deliver the intervention.
6. How	The fall risk assessment and tailored fall prevention and physical activity plan will be delivered during one face to face interview. Health coaching will be delivered via telephone or email contact.
7. Where	The intervention will be delivered to community dwelling people in Sydney and surrounds, Australia.
8. When and how much	The face to face assessment and interview will occur at the beginning of the intervention period and will last for approximately 2 hours. The telephone-based health coaching will occur after the face to face assessment and interview, once every 2 weeks for approximately 20 minutes for a total duration of 6 months.
9. Tailoring	The fall prevention aspect of the intervention will be tailored to individual need with reference to the fall risk assessment results. The physical activity plan will be tailored to participant goals, current physical ability and preferences.

### Control group

The control group (n = 65) will receive the same booklet “Staying Active and On Your Feet” and will be advised to continue their usual activities including any health service contact, so control group participants will not be disadvantaged by being in the study. At the conclusion of the trial, control group participants will be assisted to find physical activity opportunities in their local area through access to the NSW Ministry of Health *Active and Healthy* website.

### Outcomes

#### Primary outcomes

The four primary outcomes will be: physical activity participation, expressed as mean counts/minute/day, assessed over a 7-day period using a matchbox-sized accelerometer (*ActiGraph* GT3X+), measured at both six and 12 months post-randomisation; and mobility-related goal attainment, assessed using the Goal Attainment Scale (GAS), measured at both six and 12 months post-randomisation [16].

*ActiGraph* GT3X+ is the most researched accelerometer in the physical activity and public health field over the last 15 years and has been shown to be a valid instrument [17]. Participants will be instructed to wear the accelerometer on the right hip, attached via an adjustable elastic belt, for 7 consecutive days during waking hours (except during water-based activities or bathing). Activity counts per second will be collected at a sampling frequency of 30Hz and reintegrated to 60-second epochs for data analysis. The mean counts/minute/day *ActiGraph* measure will be computed as the total counts accumulated in a valid day divided by the wear time of that day. To be considered as a valid day for analysis, *ActiGraph* wear time must include 10 hours or more. Periods of 90-minutes or more of consecutive zeros (indicating non-use) will be considered as non-wear time. Accelerometer data will be manually checked against participant diaries/calendars to verify wear time and erroneous data will be excluded prior to analysis. Physical activity participation will be assessed at both six and 12 months after participant randomisation, and *ActiGraph* data will be extracted by a research assistant who is unaware of group assignment (i.e. blinded outcome assessment).

Two mobility-related goals will be established at baseline by all participants using the GAS with assistance from a research assistant or health coach. Once the goals are agreed upon the research assistant or health coach and participant will then predict the GAS outcomes on a five-point scale ranging from −2 to +2, where a score of 0 indicates achievement of the set goal, a score of −1 indicates no change from the baseline level of ability for that goal type, −2 indicates worse performance than at baseline and +1 and +2 indicate ‘somewhat better’ and ‘much better’ performance than the set goal, respectively.

Attainment of the agreed mobility-related goals will be assessed at both six and 12 months after participant randomisation by a research assistant who is unaware of group assignment.

#### Secondary outcome measures

The secondary outcomes will be: falls, recorded with monthly postal calendars over a period of 12 months; the proportion of people meeting the physical activity guidelines of 150 minutes per week of moderate to vigorous physical activity (MVPA) (intensity defined using the Freedson equation of ≥ 1952 counts/minute from the *Actigraph* to permit comparisons with other studies [18] and we will also examine this outcome using a cut point of 1040 counts/minute which is calibrated to detect MVPA (i.e., ≥ 3.7 METs) in older adults [19]); quality of life, assessed with the EQ-5D [20]; fear of falling, assessed using the short-form Falls Efficacy Scale International [21]; mood, assessed with the positive subscale of the Positive Affect Scale [22]; mobility limitation, assessed using the Late Life Function and Disability Index [23]. All secondary outcomes will be measured at both 6 and 12 months after randomisation with the exception of falls, which will only be measured at 12 months after randomisation. The intervention group will also complete a brief survey of barriers and enablers to ongoing physical activity participation at the 6 month time point.

### Analysis of outcomes

Accelerometer data will be analysed using *ActiLife 6* software. Acceptable wear time will be set a priori and defined as 4 days or more of 10 hours or more per day. General linear models will be used to assess the effect of group allocation on the continuously-scored primary (average physical activity counts per minute) and secondary outcome measures (quality of life, fear of falling, mood, mobility limitation), at both six and 12 months after randomisation, after adjusting for baseline scores.

Between-group differences in mobility-related goal attainment, at both six and 12 months after randomisation, will be analysed with ordinal regression. To aid interpretation of the GAS, the scores will also be dichotomised (goal met versus goal not met), and odds ratios calculated.

The number of falls per person-year will be analysed using negative binomial regression models to estimate the between-group difference in fall rates after one year (secondary outcome). Modified Poisson regression models will be used to compare groups on dichotomous outcome measures (proportion of fallers, proportion meeting physical activity guidelines). All analyses will be pre-planned, conducted while masked to group allocation and will use an intention-to-treat approach.

### Sample size justification

A total of 130 participants (65 per group) will provide 80% power to detect a 15% between-group difference in the primary physical activity outcome (i.e., a between-group difference of 35 mean counts per minute during wear time, standard deviation of 91), a dropout rate of 15% and alpha of 5%. The estimates of mean accelerometer counts per minute for this calculation were taken from the 263 community dwelling USA-based women aged 65 and older in a large sample of accelerometer data [24]. We took a conservative approach and estimated the proportion of dropouts at 15% although our previous trials have had lower dropout rates. These calculations were undertaken in Stata 12 using the sampsi command, assuming a 0.7 correlation between measures and assuming a post-test between-group comparison that adjusted for baseline scores.

A sample size of 130 will also provide 80% power to detect a clinically meaningful 20% between-group difference in goal attainment scores. The sample size will also be sufficient to detect between-group differences in the order of 10-15% for the secondary outcome measures.

### Ethics and dissemination

The trial protocol has been approved by the Human Research Ethics Committee at The University of Sydney, Sydney, Australia (approval number 2013/789). The results of this trial will be disseminated via peer reviewed journal articles, presentations at international conferences and participants newsletters.

## Discussion

This trial is highly significant given the dual importance of falls and inactivity for individuals and health care systems. Public health recommendations for older adults highlight the need to engage in a combination of aerobic, muscle strength, flexibility and balance activities. This trial is the first to offer an integrated strategy that can fulfil this goal. Population surveys of older adults indicate very low participation in fall prevention balance exercise [25] with the vast majority engaged in aerobic physical activity, such as walking, but this has not been demonstrated to prevent falls [26].

Hence, this trial will address a key gap in the current evidence regarding physical activity and fall prevention for older people. It will provide a model for an integrated falls and physical activity assessment and intervention program that could be directly implemented within Australian health services. The trial findings will be disseminated in peer-reviewed journals, and through scientific and professional conferences.
